# Anterior Poliomyelitis

**Published:** 1910-11

**Authors:** Theo Toepel

**Affiliations:** Atlanta, Ga.


### ANTERIOR POLIOMYELITIS.

By Dr. Theo Toepel, M. D., Atlanta, Ga.

*Etiology.*—Anterior Poliomyelitis (myelitis of the Anterior Horns) is usually considered to be peculiarly an affection of childhood and infancy, and, hence, is generally termed infantile spinal palsy—the name given it in 1840 by Heine. It was not until 1873 that the first adult case was described by Gombault, and three more by Bernhardt in 1874, two of which cases had paralysis of all four extremities.

The mortality and severity of the disease would seem to be higher in adult cases. During Dr. Taylor's four years and three months' service at the Ont. patient Department of Mass. General Hospital, 76 cases were diagnosed as adult anterior poliomyelitis, 10 of which were upward of twelve years and five of which were eighteen years and more.

The relative frequency of the affection in childhood mav be inferred from the statement of Holmes Coote, that out of 1,000 children in the Royal Orthopedic Hospital, 80, or 8 per cent., suffered from infantile palsy. The great majority of cases occur between the ages of six months and three years. It is the most frequent spinal cord disease in children, an acute transverse myelitis rarely occurring before the tenth year, and then not so frequently as anterior poliomyelitis.

In a tabulation of cases collected by five observers, Starr finds that 472 out of 609 cases, or 77 per cent., occur before the fourth year; 420, or 60 per cent., before the third year. Of 83 cases, collected by Schultze, there were 11 during the first year, 31 during the second year, and 3 over five years, and in 250 cases collected by Heine, Duchenue the younger, and Barlow, 154 were tween six months and two years. One case occurred at 63. Sex has no influence whatever upon its production; statistics show a fraction in favor of the boys. The disease is almost as frequent among the children of the wealthy as among those of the poor. Heredity does not seem to have any influence. The disease occurs with equal frequency in city and country.

The most conspicuous factor in the causation of this disease in children, is the season of the year—a fact first emphasized by Ginkler some years ago. 78.3 per cent. were attacked in the hot months of the year, that is, from May to September, as reported by this authority.

The exact relationship between digestive disturbances and the occurrence of anterior poliomyelitis is difficult of determination, as the attack at times occurs with an initial vomiting and gastric crisis.

Cases are frequently attributed to a fall, and traumatic hemorrhage into the substance of the cord, and symptoms resembling this affection may and do occur; but in the majority of cases in children, inasmuch as falling is ordinarily a common symptom of the onset, and the interval between the particular fall and the onset of the disease is great, the association is of no importance.

General epidemics have been reported.

*Pathology.*—The morbid process of anterior poliomyelitis consists of an acute destructive inflammatory lesion, mainly, though not exclusively, confined to the large multiplar cells of the anterior cornua in the gray matter of the spinal cord, and their neuraxous passing out through the anterior roots, and the secondary degenation and destruction of nerve-cells and marked changes in the peripheral nerves. In some severe cases there is a dorsal involvement, but in the large majority of cases the disease is confined to the cervical and lumbar enlargements of the cord.

*Symptoms.*—This disease is usually seen by the orthopedic surgeon when atrophy, crippling deformities, and contractures present a comprehensive picture of the chronic stage; but to appreciate this affection thoroughly, it is necessary to mention the early stages also.

The course of the disease is most conveniently divided into four stages: (1) the initial stage; (2) stationary period, which lasts from a day to a month; (3) a period of "regression," which lasts from one to six months, during which the motor function returns to certain of the affected muscles; (4) a chronic stage, during which the degenerative changes and contractures occur, and during which slight and gradual improvement may intervene at any period.

1. The initial stage is ushered in with moderate fever (102 F.), general irritability, aching pains in the loins, joints and limbs, convulsions, vomiting, diarrhea, muscular twitchings, and great cerebral disturbance—headache, drowsiness, or even stupor. Multiple neuritis may be associated, and may form aggravating complication by its persistence. The pyrexia, though rarely high, may reach 105° or 106° F. The general cerebral disturbance is so marked as to have been attributed to meningitis by both layman and practitioner. The paralysis is monoplegic in its distribution in nearly half the cases, and is much more frequent in the lower than in the upper extremity.

2. Omit discussion.

3. Omit discussion.

4. The chronic stage, characterized by deformities and dislocations from atrophy and contractures, is of peculiar interest to the orthopedic surgeon. Six months after the onset of the disease marks the limit of rapid improvement, and the improvement which may go on for years, is always slow and very slight after this period. At this time may be estimated pretty accurately the amount and distribution of the permanent paralysis, which is always much less than at first appeared. This permanent paralysis may affect only a single muscle, or may include all the extremities and some of the trunk muscles.

*Diagnosis.*—The symptoms in well established infantile paralysis are so strikingly peculiar that a diagnosis is not difficult, and yet in the early stages, before the development of the paralysis, a correct diagnosis is more difficult to establish. The pyrexia, convulsions and vomiting are frequently mistaken for the prostration following some acute affection. The characteristic symptoms upon which the practitioner must rely for a correct establishment of a diagnosis are: 1. Sudden onset. 2. Motor paralysis, tending toward regression. 3. Lost or diminished reflexes. 4. Paralyzed muscles are at all times flaccid. 5. Change of electromotor reaction. 6. Atrophy and deformities.

*Prognosis.*—With modern therapeutic, mechanical, and operative means, the prospects of improvement in this affection are now exceedingly promising. Treatment faithfully and persistently continued is frequently rewarded by the return of power and usefulness in an atrophied and helpless limb, while apparatus and surgical skill will not only correct deformities, but will hasten and increase the improvement.

*Treatment.*—Medical, at stage of onset. The indications are to reduce the temperature, to remove the exciting cause, and to relieve the urgent symptoms present. A purge of calomel, rhubarb, or magnesia, should be given. A general hot bath should be administered, and the child placed on the side or face to limit stasis of blood in the cord; mustard plasters, tincture of iodine, or dry cups should be applied to the spine, especially over the cervical and lumbar enlargements. For dental irritation the gums should be lanced at once. If genital irritation exists and phimosis be suspected as the cause, circumcision will be indicated as soon as the more acute symptoms have subsided, and may be followed by the speedy amelioration of the paralysis.

*Stage of Paralysis.*—During the early period of paralysis the parts are to be protected from strain and pressure, and measures are to be taken which will diminish the amount of the deformity, hasten the regression, and secure, if possible, the maximum improvement. The affected parts are carefully wrapped in cotton batting to keep up body temperature. Massage from the gentle stage is gradually increased to ordinary massage.

Exercise of the paralyzed limb, preferably on the principles of the Swedish movement cure, will form a valuable adjunct to the massage. Muscle and brain are developed by recriprocal action, and every movement of a composite nature develops equally the gray centers of the brain and the cord. Active muscular exercise, however obtained, enables the weakened muscles to regain their power and daily places them at a great advantage.

Electricity is the most important remedy at our command to restore power to the paralyzed muscles. Its use should always be deferred until all symptoms of inflammation have subsided.

*Mechanical Treatment.*—This must support and protect the limb in a manner which will render it most serviceable for progression, and at the same time exercise as much as possible the weakened muscles, and prevent and correct deformities.

*Operative Treatment.*—This is demanded when mechanical methods are inefficient or undesirable, and affords the most speedy and satisfactory result in the severest cases. It includes tenotomy, tendon shortening, transplantation of muscles and tendons, aponeurotomy, myotomy, neural anastomosis, forcible straightening, excision, osteatomy, arthrodesis and amputation.

